# Gut Microbiota Predicts Healthy Late-Life Aging in Male Mice

**DOI:** 10.3390/nu13093290

**Published:** 2021-09-21

**Authors:** Shanlin Ke, Sarah J. Mitchell, Michael R. MacArthur, Alice E. Kane, David A. Sinclair, Emily M. Venable, Katia S. Chadaideh, Rachel N. Carmody, Francine Grodstein, James R. Mitchell, Yangyu Liu

**Affiliations:** 1 Channing Division of Network Medicine, Brigham and Women’s Hospital and Harvard Medical School, Boston, MA 02115, USA; spske@channing.harvard.edu (S.K.); phfrg@channing.harvard.edu (F.G.); 2 State Key Laboratory of Pig Genetic Improvement and Production Technology, Jiangxi Agricultural University, Nanchang 330045, China; 3 Department of Molecular Metabolism, Harvard T.H. Chan School of Public Health, Boston, MA 02115, USA; macarthur@g.harvard.edu; 4 Department of Health Sciences and Technology, ETH Zurich, 8005 Zurich, Switzerland; james.mitchell@hest.ethz.ch; 5 Paul F. Glenn Center for Biology of Aging Research, Department of Genetics, Blavatnik Institute, Harvard Medical School, Boston, MA 02115, USA; alice_kane@hms.harvard.edu (A.E.K.); david_sinclair@hms.harvard.edu (D.A.S.); 6 Department of Human Evolutionary Biology, Harvard University, Cambridge, MA 02138, USA; emilyvenable@g.harvard.edu (E.M.V.); kchadaideh@g.harvard.edu (K.S.C.); carmody@fas.harvard.edu (R.N.C.); 7 Department of Epidemiology, Harvard T.H. Chan School of Public Health, Boston, MA 02115, USA

**Keywords:** gut microbiota, mice, calorie restriction, healthy aging, machine learning

## Abstract

Calorie restriction (CR) extends lifespan and retards age-related chronic diseases in most species. There is growing evidence that the gut microbiota has a pivotal role in host health and age-related pathological conditions. Yet, it is still unclear how CR and the gut microbiota are related to healthy aging. Here, we report findings from a small longitudinal study of male C57BL/6 mice maintained on either *ad libitum* or mild (15%) CR diets from 21 months of age and tracked until natural death. We demonstrate that CR results in a significantly reduced rate of increase in the frailty index (FI), a well-established indicator of aging. We observed significant alterations in diversity, as well as compositional patterns of the mouse gut microbiota during the aging process. Interrogating the FI-related microbial features using machine learning techniques, we show that gut microbial signatures from 21-month-old mice can predict the healthy aging of 30-month-old mice with reasonable accuracy. This study deepens our understanding of the links between CR, gut microbiota, and frailty in the aging process of mice.

## 1. Introduction

The proportional population of older persons is growing across the globe [1]. This demographic shift will increase the prevalence of age-related disease and place a significant burden on health costs and social care. Moreover, increased longevity (i.e., lifespan) does not necessarily translate to better quality of life (i.e., healthspan) [2]. Thus, it is imperative to improve our understanding of mechanisms underlying aging processes and develop practical interventions to promote healthy aging and delay age-related diseases.

Aging is one of the most complex biological processes that affects a wide array of physiological, genomic, metabolic, and immunological functions [3,4]. These age-related functional changes can lead to organ and systemic decline, which ultimately results in death. There is now growing evidence that the gut microbiota interacts with these physiological functions, and thereby plays a pivotal role in host health and age-related pathological conditions [5,6,7]. The gut microbiota is regulated by a complex interplay between host and environmental factors, including age, diet, antibiotics, genetics, and lifestyle [8,9]. In turn, changes in the gut microbiota can alter host physiology, increasing the incidence and/or severity of many diseases that contribute to morbidity and mortality in later life, such as inflammatory bowel disease [10], type 2 diabetes [11], obesity [12], cardiovascular disease [13], and neurodegenerative disease [14]. During host aging, the gut microbiota undergoes dramatic changes in composition and function [15,16,17,18,19]. The gut microbiota of elderly people is different from that of adults [17,20,21], and microbial compositions in the elderly correlate with measures of frailty, barrier dysfunction, gut motility, and inflammation [22]. Nevertheless, the extent to which these changes result from host aging or contribute to it remains unclear. Unlike other organs, the gut microbiota might not be expected to follow the same general trajectory of somatic senescence [23]. 

**Figure 1:**
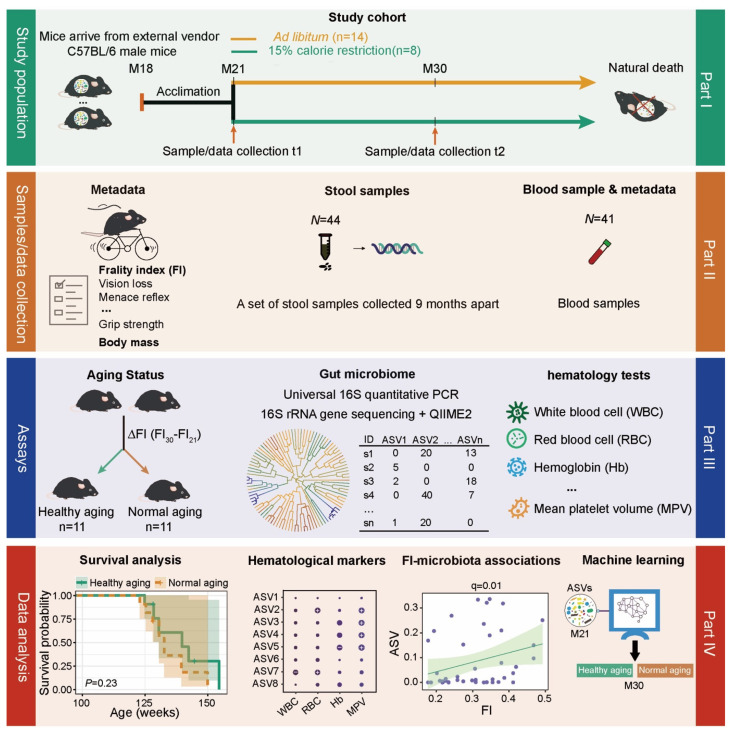
Schematic diagram showing the experimental design. The study cohort was comprised of 22 adult male C57BL/6 mice, which were recruited into the study at 21 months of age after having been maintained since birth under standard husbandry conditions (see Methods). We collected blood and fecal samples and measured frailty using a compound index at 21 months (baseline) and 30 months of age. Following baseline measurements, we randomly divided these mice into two diet groups, fed either *ad libitum* (AL, *n* = 14) with standard chow or under mild (15%) calorie restriction (CR, *n* = 8). Mice were then followed longitudinally until death. We performed universal 16S quantitative PCR (qPCR) to quantify absolute bacterial abundance and 16S rRNA gene sequencing to determine taxonomic composition, using QIIME2 to characterize the ASV microbial features. Blood markers were measured using standard methods. We then used the median FI change (denoted as ΔFI) between 21 and 30 months of age to delineate healthy versus normal aging.

Calorie restriction (CR), a dietary regimen that reduces the consumption of food without resulting in malnutrition, has been shown in animal models to retard development of age-related chronic diseases and extend the lifespan [24,25,26,27]. In addition to effects on host physiology, CR can also reshape the gut microbial community in both humans [28,29] and animal models [30,31,32]. CR-induced alterations to the gut microbiome might play a role in extending lifespan and healthspan and delaying the onset of age-related disorders. In this study, we evaluate how the gut microbiota changes during the aging process in mice and test whether gut microbial features can predict healthy aging (Figure 1, see Methods for details). To do this, we performed quantitative PCR (qPCR) targeting the 16S rRNA gene and 16S rRNA gene sequencing of bacterial DNA extracted from fecal samples from a cohort of aging male mice tracked from 21 months of age. We investigated associations between these microbial signatures and biomarkers of host condition, including weight, food intake, hematological markers, and frailty index (FI), a validated biomarker of biological age that is a strong predictor of mortality, morbidity, and other age-related outcomes [33]. Examining how signatures in the gut microbiota predict future aging status can illuminate the utility of the gut microbiota as an early indicator of healthy aging.

## 2. Results

### 2.1. The Association of the Physiological Characteristics with Chronological Age

**Figure 2:**
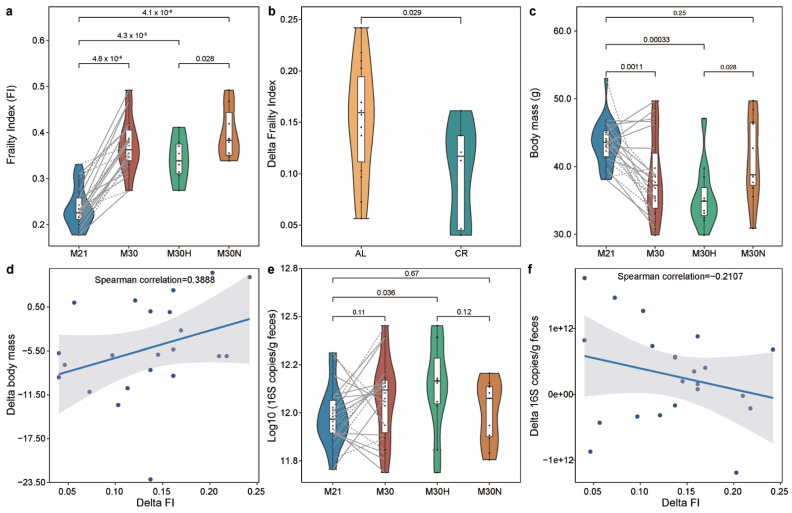
Frailty index associates with chronological age in mice. (**a**) Frailty index changes with age. Mice at 30 months of age were grouped into healthy and normal aging based on the median ΔFI. (**b**) The effect of caloric restriction on the ΔFI between 21 and 30 months of age. (**c**) Comparison of body mass (BM) for different groups. (**d**) The association between ΔFI and ΔBM in all mice. (**e**) Comparison of total bacterial load for different groups. (**f**) The association between ΔFI and ΔBL in all mice. Points obtained for the same subject from 21 and 30 months of age are joined by solid (AL diet) and dotted (CR diet) lines. *p*-value shown in (**a**–**c**,**e**) are the result of a Wilcoxon–Mann–Whitney test (unpaired) and a Wilcoxon signed rank test (paired). The correlation coefficient shown in (**d**,**f**) is the result of a Spearman correlation. The lines show lm fit for the data, and shaded areas show 95% confidence intervals for the fit.

The mouse clinical frailty index (FI) is based on established clinical signs of deterioration in mice [34,35]. Briefly, the clinical assessment includes evaluation of the integument, the musculoskeletal system, the vestibulocochlear/auditory systems, ocular and nasal systems, digestive system, urogenital system, respiratory system, signs of discomfort, body weight, and body surface temperature. FI score is continuous from 0–1, with higher values indicating worse frailty. A cutoff of 0.21 has been previously used in rodents [36] to stratify frailty as either high (frail: FI ≥ 0.21) or low (not frail: FI < 0.21). But as mice reached 30 months old in our study, they all became frail with higher FI score (FI > 0.21). Indeed, as shown in Figure 2a and Figure S1a, FI score significantly increased with chronological age from 21 to 30 months at the population level (*p*-value = 4.8 × 10^−6^, Wilcoxon signed-rank test). Hence, instead of using a fixed FI score cutoff, we instead used the median value of FI change (denoted as ΔFI) to delineate healthy versus normal aging. Specifically, we calculated ΔFI between month 21 and 30 for each mouse, and then we dichotomized those mice at month 30 into two groups based on the median value of their ΔFI: ‘healthy aging’ (age in weeks: mean 121.78 ± standard deviation 3.88; ΔFI: 0.088 ± 0.038; FI: 0.342 ± 0.048; *n* = 11); and ‘normal aging’ (age in weeks: 121.42 ± 4.07; ΔFI: 0.179 ± 0.034; FI: 0.398 ± 0.055; *n* = 11). CR diet was associated with a lower level of ΔFI at month 30 than AL diet (Figure 2b, *p*-value = 0.029, Wilcoxon–Mann–Whitney test). In particular, 87.5% (7/8) of mice with CR diet belonged to the healthy aging group compared to just 36.4% (4/11) of mice fed *ad libitum*. These results suggest that CR had a beneficial effect on aging, consistent with previous studies [25]. 

We found that the body mass (BM) of mice generally decreased during aging (Figure 2c, *p*-value = 0.0011, Wilcoxon signed-rank test), an effect contributable to healthy aging mice due to the fact that most of them (63.64%) were from the CR group (Figure S1b). At 30 months of age, the BM of the healthy aging mice was significantly lower than the normal aging (Figure 2c, *p*-value = 0.028, Wilcoxon–Mann–Whitney test) and baseline mice (Figure S1b, *p*-value = 0.0049, Wilcoxon signed-rank test). To better understand this finding, we calculated delta change of BM (ΔBM) between month 21 and 30 for each mouse. The ΔFI was positively associated with ΔBM (Figure 2d, *ρ* = 0.3888, Spearman correlation), suggesting that a normal aging mouse (with large ΔFI) is associated with an increasing level of BM. In addition, we found that the BM in healthy aging mice gradually decreased over time (Figure S2a), especially in those mice with CR diet (Figure S2b). Moreover, normal aging mice showed rapid loss of BM after some time points (Figure S2). Using Kaplan–Meier survival analysis, the differences in cumulative survival rates were not statistically significant between healthy and normal aging mice (Figure S3, *p*-value = 0.23, log-rank test). However, the healthy aging mice showed qualitatively longer lifespan (134.36 ± 9.43) than normal aging (131.06 ± 7.53) mice (*p*-value = 0.313, Wilcoxon–Mann–Whitney test), as some mice from the healthy aging group lived substantially longer.

### 2.2. Aging-Related Changes in Gut Microbial Community

Using universal 16S qPCR, we first measured the total bacterial load (BL) in the stool samples (Figure 2e and Figure S1c). The results showed the total BL detected in healthy aging mice was higher than the BL present in the normal aging mice (Figure 2e). For the changes of total BL over time (ΔBL), we found ΔFI was inversely associated with ΔBL (Figure 2f, *ρ* = −0.2107, Spearman correlation), suggesting that a normal aging mouse (larger ΔFI) is associated with a decreasing total BL.

**Figure 3:**
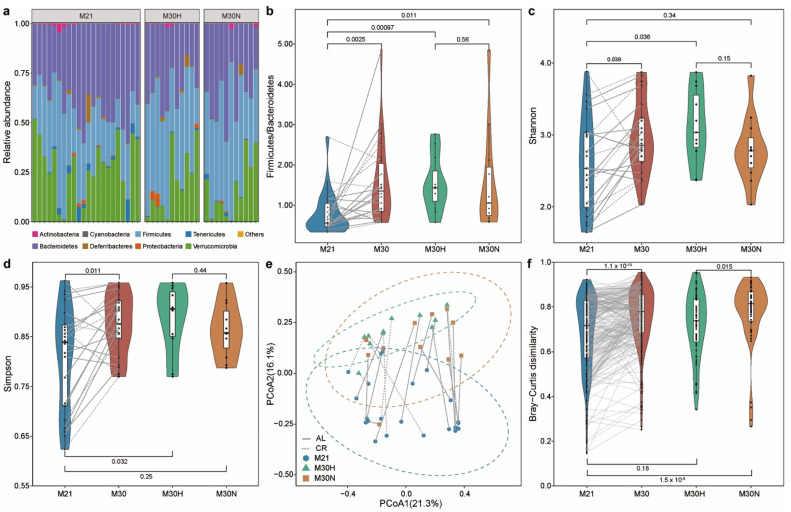
Impact of aging on gut microbial communities. (**a**) Relative abundance of bacterial phyla. (**b**) The ratio of Firmicutes to Bacteroidetes. Alpha diversity using the Shannon (**c**) and Simpson (**d**) index. (**e**) Beta diversity using principal coordinate analysis (PCoA) of Bray–Curtis dissimilarity. The dotted ellipse borders with color represent the 95% confidence interval. (**f**) Boxplot of gut microbiota Bray–Curtis dissimilarity between subjects within each group. Points obtained for the same subject from 21 and 30 months of age in (**b**–**e**) are joined by solid (AL diet) and dotted (CR diet) lines. Points obtained for the same subject pairs from 21 and 30 months of age in (**f**) are joined by solid line. *p*-value shown in (**b**–**d**,**f**) are the result of a Wilcoxon–Mann–Whitney test (unpaired) or Wilcoxon signed rank test (paired).

We then measured the gut microbial community compositions of those stool samples using 16S rRNA gene sequencing (see Methods, Table S1). Phylum-level taxonomic profiles of the gut microbiome samples of those mice are shown in Figure 3a. Consistent with previous studies [37,38], we found that Bacteroidetes, Firmicutes, and Verrucomicrobia were the most dominant phyla in the murine gut microbiota. Notable age-related compositional shifts included an enrichment in Firmicutes, and reduction in Bacteroidetes and Verrucomicrobia, although such trade-offs among dominant phyla are expected *a priori* in relative abundance data. Moreover, the Firmicutes/Bacteroidetes ratio of the gut microbiota increased with age (Figure 3b, *p*-value = 0.0025, Wilcoxon signed-rank test). Both healthy aging and normal aging mice showed higher values for this ratio compared with baseline mice (Figure S4a). 

Using the Shannon and Simpson indices as alpha diversity measures, we found that alpha diversity increased with age (Figure 3c,d and Figure S4b,c), consistent with a previous mouse study [39]. Interestingly, we found that the Shannon diversity was only significantly higher in healthy aging mice compared to baseline mice (Figure S4b, *p*-value = 0.019, Wilcoxon signed-rank test). In addition, a clear separation (permutational multivariate analysis of variance (PERMANOVA) test, *p*-value = 0.0001, Bray–Curtis dissimilarity) could be seen between mice at 21 and 30 months of age in the principal coordinate analysis (PCoA) plot based on Bray–Curtis dissimilarity (Figure 3e). Indeed, PERMANOVA test indicated significantly altered microbial compositions for both healthy aging (*p*-value = 0.0004) and normal aging (*p*-value = 0.0086) mice between baseline and 30 months of age (Figure S4d). However, we found no significant difference between healthy aging and normal aging mice at both 21 (*p*-value = 0.8747) and 30 (*p*-value = 0.3536) months of age. Bray–Curtis dissimilarity was higher among individuals within normal aging mice compared to baseline mice (Figure S4e, *p*-value = 4 × 10^−8^, Wilcoxon signed-rank test) or healthy aging mice (Figure 3f, *p*-value = 0.015, Wilcoxon–Mann–Whitney test). This suggests that normal aging is characterized by high variations in gut microbiota between individuals. 

### 2.3. The Effect of Aging on Hematology and Associations between Gut Microbiota and Blood Markers

Aging is associated with a decline in immune system function at multiple levels [40]. To explore aging-related immune system modifications, we measured hematological parameters over time (Table S2). We found that the mice at 30 months of age tended to have higher level (with *p*-value < 0.05) of neutrophils percentage, neutrophil to lymphocyte ratio (NLR), monocytes percentage (MOp, % of leukocytes), red cell distribution width (RDW, % variation), and mean platelet volume (MPV, fL), but lower level (*p*-value < 0.05) of white blood cells (WBC, k/uL), lymphocytes (LY, k/uL), lymphocytes percentage (LYp, % of leukocytes), red blood cell (RBC, M/uL), hemoglobin (Hb, g/dL), mean corpuscular volume (MCV, fL), and hematocrit (HCT, % volume) when compared with mice at 21 months of age. Notably, higher NLR levels (an important biomarker of systemic inflammation [41]) levels in 30-month-old mice were mainly observed in normal aging mice (*p*-value = 0.016). These results confirm prior observations that high levels of inflammation are not an inevitable consequence of aging, but are rather associated with normal or unhealthy aging. Moreover, at 30 months of age, we found that normal aging mice had significantly higher MPV but normal PLT.

**Figure 4:**
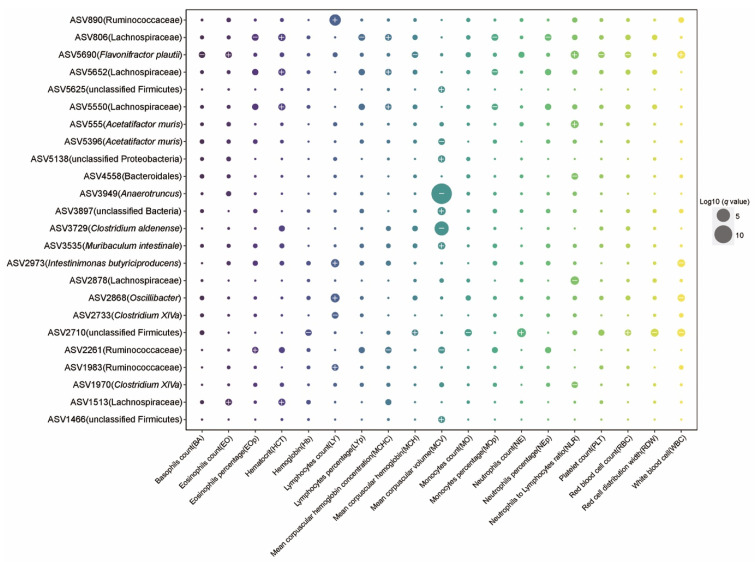
Identification of associations between blood cell and gut microbial features. Dot plot showing the links between the blood markers and gut microbial taxa identified using MaAsLin2. The sizes of dots represent the *q*-values from MaAsLin2. The greater the size, the more significant the association. Symbols indicate the directions of associations in a given model: plus, significant positive associations; minus, significant negative associations. Threshold for the FDR-corrected *q*-value was set at 0.2. Linear mixed effects models were applied to the association with each mouse’s identifier treated as set as a random effect.

Given the effects of aging process on hematology, we next used MaAsLin2 (multivariate analysis by linear models) [42] to evaluate the associations between microbial taxa and blood markers. These linear mixed models accounted for within-individual correlation from the study’s repeated sampling design, as well as occasional missing observations at some time points. To control for potential confounding variables, we added four covariates into the model as fixed effects, including diet treatment, cohort, cage, and body mass. In addition, each mouse’s identifier was treated as a random effect. A total of 24 ASVs (amplicon sequence variant) features were significantly associated with at least one blood marker (Figure 4, *q*-value ≤ 0.2, Table S3). In general, blood markers correlating most with microbial taxa included MCV, LY, and NLR. For example, MCV was inversely associated with the abundance of ASV 3949 (*Anaerotruncus, q-*value = 2.38 × 10^−14^) and ASV3729 (*Clostridium aldenense, q*-value = 1.52 × 10^−6^), and LY was positively associated with ASV890 (Ruminococcaceae, *q*-value = 0.0004), ASV2868 (*Oscillibacter*, *q*-value = 0.015), and ASV2973 (*Intestinimonas butyriciproducens, q*-value = 0.035). NLR was positively associated with ASV5690 (*Flavonifractor plautii*, *q*-value = 0.04) and ASV555 (*Acetatifactor muris*, *q*-value = 0.048), and negatively associated with ASV2878 (Lachnospiraceae, *q*-value = 0.028), ASV4558 (Bacteroidales, *q*-value = 0.146), and ASV1970 (*Clostridium XlVa*, *q*-value = 0.189).

### 2.4. Microbial Taxa Related to Frailty Index and Healthy Aging

**Figure 5:**
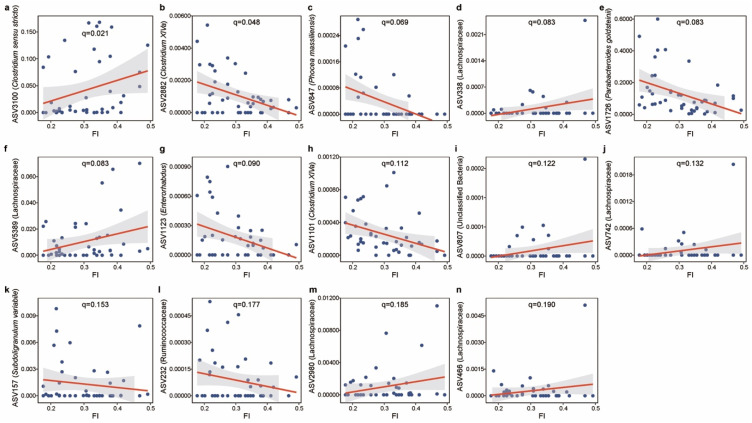
The significant associations between FI and gut microbial features. (**a**) ASV3100 (*Clostridium sensu stricto*). (**b**) ASV2882 (*Clostridium XlVa*). (**c**) ASV847 (*Phocea massiliensis*). (**d**) ASV338 (Lachnospiraceae). (**e**) ASV1726 (*Parabacteroides goldsteinii*). (**f**) ASV5389 (Lachnospiraceae). (**g**) ASV1123 (*Enterorhabdus*). (**h**) ASV1101(*Clostridium XlVa*). (**i**) ASV807 (Unclassified Bacteria). (**j**) ASV742 (Lachnospiraceae). (**k**) ASV157 (*Subdoligranulum variabile*). (**l**) ASV232 (Ruminococcaceae). (**m**) ASV2980 (Lachnospiraceae). (**n**) ASV466 (Lachnospiraceae). Data shown are the relative abundance versus FI for ASVs that were significantly associated with FI in MaAsLin2. Threshold for the FDR-corrected *q*-value was set at 0.2. Linear mixed-effects models (LMMs) were applied to the association with each mouse’s identifier treated as a random effect. The lines show lm fit for the data, and shaded areas show 95% confidence intervals for the fit.

We next investigated the FI in relation to the microbial features using MaAsLin2, in which diet, cohort, cage, and body mass were included as fixed effects and each mouse’s identifier was included as a random effect. We observed a set of 14 microbial features that were strongly linked to FI (Figure 5, *q-*value ≤ 0.2, Table S4). Consistent with previous reports that the abundance of the *Clostridium sensu stricto* genus increases with aging [43,44,45], ASV3100 (*Clostridium sensu stricto*: *q*-value = 0.021) was positively associated with the FI. *Clostridium XlVa* [46] (ASV2882, *q*-value = 0.048 and ASV1101: *q*-value = 0.112) and *Subdoligranulum variabile* [47] (ASV157, *q*-value = 0.153), known as important producers of butyrate, were found to be negatively associated with FI. We also found inverse associations of the FI with taxa such as ASV847 (*Phocea massiliensis*, *q*-value = 0.069), ASV 1726 (*Parabacteroides goldsteinii*, *q*-value = 0.083), and ASV1123 (*Enterorhabdus*, *q*-value = 0.090). A previous study linked *Parabacteroides goldsteinii* with a reduction of intestinal inflammation and enhancement of cellular mitochondrial and ribosomal activities in the colon [48].

To examine potential gut microbial signatures of late-life aging, we performed differential abundance analysis using ANCOM [49] (analysis of composition of microbiomes). ANCOM identified multiple gut microbiota signatures that were significantly different between baseline and 30 months of age in healthy aging (Figure S5a and Table S5) and normal aging (Figure S5b and Table S6) mice. Most of these features were also identified when comparing all mice between 21 and 30 months of age as a group (Figure S6 and Table S7). Intriguingly, we found seven ASVs that significantly and concordantly increased with age in both healthy aging and normal aging groups (Figure S5), including ASV5550 (Lachnospiraceae), ASV5652 (Lachnospiraceae), ASV806 (Lachnospiraceae), ASV5435 (*Muribaculum intestinale*), ASV5628 (*Muribaculum intestinale*), ASV3370 (*Muribaculum intestinale*) and ASV3224 (*Clostridium cocleatum*), hinting at a universal murine microbial signature of aging. To assess how the microbial features associate with healthy aging, we calculated the differential abundance of features between healthy aging and normal aging groups at both 21 and 30 months of age (Figure S7). Our data found six (Figure S7a, Table S8) and nine (Figure S7b, Table S9) ASVs that were significantly associated with aging status at baseline and 30 months of ages, respectively. In particular, a set of microbial features were significantly enriched in healthy aging mice at 30 months of age, for example ASV648 (*Akkermansia muciniphila*), ASV73 (Ruminococcaceae), and ASV2756 (*Acetatifactor muris*). *A. muciniphila* has been observed previously to prevent the age-related decline in thickness of the colonic mucus layer and attenuate inflammation in old age [50], although recent report have also suggested it may be associated with the progression of neurodegenerative diseases [51]. Here, this microbial feature was detected and shown to be associated with healthy aging as indexed by our metrics. Normal aging mice showed increased ASV3370 (*Muribaculum intestinale*), ASV3100 (*Clostridium sensu stricto*), ASV3939 (*Turicibacter sanguinis*), and ASV1123 (*Enterorhabdus*) compared with healthy aging mice. Consistent with the positive relationship between FI and ASV3100 (*Clostridium sensu stricto*), we found that this feature was significantly higher in the normal aging group.

### 2.5. Gut Microbiota-Based Machine Learning Model to Predict Healthy Aging

**Figure 6:**
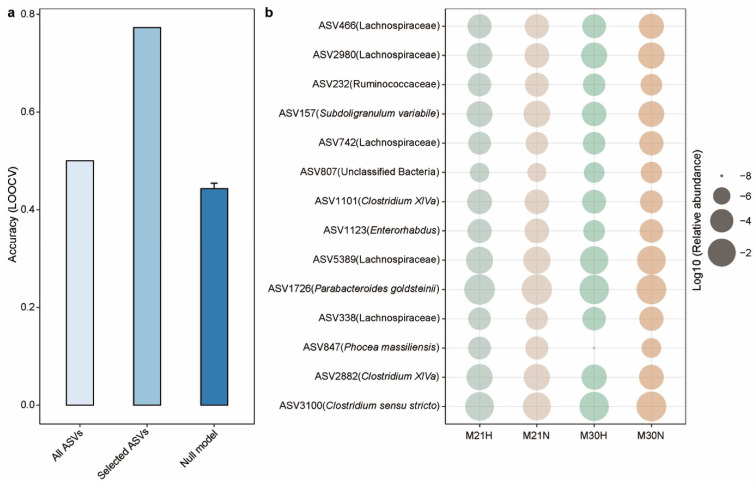
A gut microbiota-based signature moderately predicts healthy aging. (**a**) Leave-one-out (LOOCV) accuracy evaluating ability to predict healthy aging using Elastic-net (ENET). Each bar represents the performance based on different combination of microbial feature: all ASVs, 14 FI-associated ASVs, and null model with 14 randomly selected features run 100 times. Error bars represent the standard errors of the means (SEM) in null model. (**b**) The mean relative abundance of 14 FI-related ASVs across different groups. The healthy aging status at 21 months of age was determined by the aging status at 30 months of age. Relative abundances are plotted on a log10 scale.

As microbial compositions were associated with aging status, we sought to determine whether the microbial features observed in mid-life could predict healthy aging in later life. To achieve that, we employed an Elastic-net (ENET) logistic regression model to predict healthy aging. Specifically, the ENET model trained with ASVs (present in at least 10% of the samples) achieved an accuracy of 0.5 (11/22) with leave-one-out cross-validation (LOOCV) (Figure 6a). In principle, we can apply feature selection techniques to choose a subset of features from the dataset. However, to improve the biological meaning of the model, we then only selected the microbial features that significantly associated with FI. This approach included a microbial signature comprised of 14 ASVs (Figure 6b) from the gut microbiota of 21-month-old mice that exhibited power in predicting the healthy aging status of 30-month-old mice with an LOOCV accuracy of 0.773 (17/22) (Figure 6a). Notably, we also observed that *Clostridium sensu stricto* and *Enterorhabdus* were significantly overrepresented in normal aging mice at 30 months of age. A previous study found that *Clostridium sensu stricto* was significantly enriched in early onset necrotizing enterocolitis subjects [52]. *Enterorhabdus*, a member of the family Coriobacteriaceae, has been isolated from a mouse model of spontaneous colitis [53]. These findings were consistent with higher level of NLR in normal aging mice, which was used as a marker of systemic inflammation. This may partially explain the ability of these features to predict healthy aging over the subsequent 9 months. Finally, we validated our model by generating a null model with randomly selected features (number of features = 14, times = 100), which yielded a mean LOOCV accuracy of 0.443 (Figure 6a). 

## 3. Discussion

Over the last few decades, global average life expectancy has increased dramatically, resulting in a proportionately larger aging population. Currently, chronological age is the most widely used indicator of aging, yet it provides limited information on the quality of life during the aging process. Understanding how to promote healthy aging will be key to increasing healthspan. Evidence is emerging that the gut microbiota is intrinsically linked with energy metabolism and the aging process [54,55,56,57]. In this study, we observed that the mouse gut microbiota is associated with healthy aging in late-life aged mice. Moreover, we identified a specific stool-microbiota-derived signature of aging that yielded a reasonable accuracy for the prediction of healthy aging.

A better predictor of mortality and morbidity in humans than chronological age is the frailty index (FI) [58]. The FI has been reverse translated into a tool for mice that includes 31 non-invasive parameters across a range of systems [37,59]. Previous studies applied 0.21 as a cut-off point of FI to stratify between high frailty (≥0.21) or low frailty (<0.21) [36,60,61]. Given that this specific threshold provides limited insight into the aging process, we instead employed the ΔFI (FI changes between 30 and 21 months of age) to quantify the ability to maintain health conditions during aging. Indeed, those mice with higher ΔFI (based on median value) were more vulnerable and frailer. In our study, we only included the mice with basic measurements and biological samples at both 21 and 30 months, resulting in 22 male mice that were fed either AL (*n* = 14) or CR (*n* = 8) diets. To avoid the issue arising from imbalanced sample size, we stratified the mice to healthy aging and normal aging groups based on the ΔFI. As expected, 87.5% (7/8) of mice fed a CR diet belonged to the healthy aging group compared to just 36.4% (4/11) of mice fed an AL diet.

Although several previous studies demonstrated the links between gut microbiota and aging in mice, these studies mainly focused on the comparison between different growth stages [62,63,64]. In this study, we examined the gut microbiota collected at 21 and 30 months of age from 22 mice and measured the aging status. Concordant with previous reports, we found that aging was associated with increased alpha diversity [64], but in our dataset this pattern was driven by the observation of higher alpha diversity in the healthy aging cohort. Consistent with previous work [65], our study also linked aging to an increase in interindividual variation in gut microbial community composition, with interindividual variation being especially high in the normal aging group. This may suggests that the unhealthy aging-related changes in the gut microbiota are likely stochastic, leading to community instability. Our study also linked FI to several microbial features, such as ASVs from *Clostridium sensu stricto*, *Clostridium XlVa*, *Enterorhabdus*, and *Phocea massiliensis.* Importantly, we constructed a machine learning model that can predict healthy aging with an LOOCV accuracy of 0.773 (17/22) based on these FI-related microbial features. Moreover, these microbial features may be further driven by CR after 21 months of age. Indeed, we found that some predictive features (e.g., ASVs from *Clostridium sensu stricto* and *Enterorhabdus*) were only identified as differentially abundant taxa at 30 months of age. These findings suggest that key microbial taxa could potentially serve as biomarkers of aging and might contribute to the pathophysiology of aging, although the latter possibility remains to be determined.

We acknowledge the following limitations of this study. First, the sample size of the experimental cohort is relatively small and limited to male mice. Second, 16S rRNA gene sequencing limits our ability to establish associations at the strain level, suggesting that future studies with shotgun metagenomics sequencing will increase resolution. Third, the association between healthy aging and microbial taxa identified in this study does not demonstrate causality. Thus, additional research is needed to validate the mechanism behind these essential findings. Finally, the generalization of the machine learning-based gut microbial signature of aging to other murine cohorts and to humans remains unknown. However, the strengths of the study include a prospective study design, detailed phenotyping of mice, and assessment of accuracy using gut microbial features to predict healthy aging by a machine learning model.

In conclusion, we evaluated the impact of age-related changes in gut microbiota on the course of aging in late-life male mice to assess a microbiota signature associated with healthy aging. Our study suggests the possible interaction between specific gut microbiota and aging status, and motivates future work that could establish causality and the potential of future microbiota-targeted interventions to increase healthy aging. 

## 4. Methods

### 4.1. Experimental Design

Following baseline phenotypic measurements (body weight, food intake, frailty index, grip strength, and fecal collection), adult male C57BL/6 mice were randomized at 21 months of age into *ad libitum* diet (AL, *n* = 14) or mild calorie restriction diet (CR, 15% fewer calories than their peers consuming an *ad libitum* diet, *n* = 8) groups and followed longitudinally until death. From each birth cohort that we received, we randomized the mice equally into groups to avoid a strong birth-cohort effect. We repeated phenotypic measurements after 9 months (30 months of age) and recorded survival. We performed a qPCR analysis targeting the 16S rRNA gene as well as 16S rRNA gene sequencing on 44 stool samples, collected at 21 and 30 months of age, from 22 mice.

### 4.2. Study Population and Sample Collection

In our study, we only included the mice with basic measurements and biological samples at both 21 and 30 months. Mice were fed a standard chow based upon AIN-93G (Custom diet #A17101101, Research Diets, New Brunswick, NJ, USA). After 21 months of age, CR was initiated over a period of two-weeks in a step-down fashion (10% CR, 15% CR) to ensure no loss on mice as they transition to the restricted feeding paradigm. Mice were housed in a clean empty cage (no bedding) during the fecal sample collection. Fresh fecal samples (non-fasted) were collected in the morning (8.30 a.m.–11.30 a.m.) into sterile tubes and frozen at −80 °C until future analysis. 

### 4.3. The Measurement of Frailty Index

Frailty was measured using the validated 31-parameter mouse clinical frailty index as described previously [34,35]. Briefly, the clinical assessment includes evaluation of the integument, the musculoskeletal system, the vestibulocochlear/auditory systems, ocular and nasal systems, digestive system, urogenital system, respiratory system, signs of discomfort, body mass, and body surface temperature. The frailty index items including: (1) Alopecia (0.5: <25% fur loss, 1: >25% fur loss); (2) Fur color loss (0.5: <25% color change, 1: >25% color change); (3) Dermatitis (0.5: small section, 1: large multiple lesions); (4) Coat condition (0.5: not smooth/sleek, 1: very matted, ungroomed); (5) Loss of whiskers (0.5: some or color change, 1: no whiskers); (6) Kyphosis (0.5: some curvature, 1: strong curvature always); (7) Distended abdomen (0.5: slight bulge “W” shape, 1: clear bulge); (8) Vestibular disturbance (0.5: spinning when lowered, 1: head tilt at all times); (9) Cataracts/corneal opacity (0.5: small sections, 1: large areas of clouding); (10) Cataracts/corneal opacity (0.5: small sections, 1: large areas of clouding); (11) Eye discharge/swelling (0.5: minor discharge or swelling, 1: severe swelling or both eyes); (12) Microphthalmia (0.5: some shrinkage, 1: severe shrinkage or both eyes); (13) Malocclusions (0.5: excess or irregular teeth growth, 1: severe irregular growth); (14) Rectal prolapse (0.5: minor tissue protrusion, 1: severe tissue protrusion); (15) Penile prolapse (0.5: minor tissue protrusion, 1: severe tissue protrusion); (16) Mouse grimace scale (0.5: 1–2/5 items (whiskers, ears, cheeks, nose, eyes), 1: 3–5 items, doi:10.1038/nmeth.1455); (17) Piloerection (0.5: fur standing at neck, 1: fur standing on full body); (18) Tail stiffening (0: curling response, 0.5: some curling, 1: no response); (19) Gait (0: normal gait, 0.5: some abnormalities, especially on angle, 1: very abnormal gait); (20) Grip strength (0: as strong as a young mouse, 0.5: some reduction in grip, 1: no resistance against pull); (21) Body condition (0: normal, 0.5: too much or too little fat on hips, 1: hip bones very protruding or not felt at all); (22) Hearing loss (0: responds 3/3, 0.5: responds 1–2/3, 1: responds 0/3); (23) Vision loss (0: reaches from > 8 cm, 0.5: reaches from 2–8 cm, 1: reaches lower than 2 cm); (24) Menace reflex (0: responds 3/3, 0.5: responds 1–2/3, 1: responds 0/3); (25) Tremor (0: none, 0.5: some, especially on incline, 1: tremor at all times); (26) Growths (0: none, 0.5: <1 cm, 1: >1 cm or many); (27) Nasal discharge (0: none, 0.5: minor, 1: severe); (28) Diarrhea (0: none, 0.5: minor, 1: severe); (29) Breathing rate/depth (0: normal, 0.5: some slowing or irregularity, 1: very irregular, pauses); (30) Temperature score; (31) Body weight score. FI score is continuous from 0–1, with higher values indicating worse frailty [35]. For more detail see: http://frailtyclocks.sinclairlab.org/.

### 4.4. Hematology Analysis

In total, 25 µL of whole blood obtained via submandibular bleeding was combined with 1 µL of EDTA to prevent clotting. The sample was analyzed using a Hemavet 950 veterinary (Drew Scientific, Miami Lakes, FL, USA) multi-species hematology system using standard settings.

### 4.5. Estimation of Bacterial Load by Quantitative PCR

To estimate the gut bacterial load in our 44 fecal samples, we performed quantitative PCR (qPCR) targeting the 16S rRNA gene using the same primers employed for 16S rRNA gene sequencing (515F and 806R). Briefly, 2 µL of template DNA was combined with 12.5 µL PerfeCTa SYBR Green SuperMix Reaction Mix (QuantaBio, Beverly, MA, USA), 6 µL nuclease-free H2O, and 2.25 µL of each primer. Amplification was performed on a Bio-Rad CFX384 Touch (Bio-Rad, Hercules, CA, USA) in the Bauer Core Facility at Harvard University using the following cycle settings: 95 °C for 10 min, followed by 40 cycles of 95 °C for 15 s, 60 °C for 40 s and 72 °C for 30 s. Reactions were performed in triplicate with the mean value used in statistical analyses. Cycle-threshold values were standardized against a dilution curve of *Escherichia coli* genomic DNA at the following concentrations (ng/µL): 100, 50, 25, 10, 5, 1, and 0.5, plus a no-template (negative) control. Bacterial DNA concentrations were normalized to 16S copies/µL, then multiplied by the total extracted DNA volume (50 µL) and divided by the grams of fecal matter utilized in the extraction of template DNA (varied), allowing us to report gut bacterial load as 16S rRNA gene copies per gram of feces.

### 4.6. DNA Isolation and 16S rRNA Gene Sequencing

Gut microbial DNA was isolated using the DNeasy PowerSoil Pro Kit (Qiagen, Hilden, Germany) and PCR-amplified using barcoded primers targeting the V4 region of the bacterial 16S rRNA gene [515F (GTGYCAGCMGCCGCGGTAA) and 806R (GGACTACNVGGGTWTCTAAT); Integrated DNA Technologies]. The following thermocycler protocol was used: 94 °C for 3 min, 35 cycles of 94 °C for 45 s, 50 °C for 30 s, and 72 °C for 90 s, with a final extension at 72 °C for 10 min. Triplicate PCR reactions for each sample were pooled and amplification was confirmed by 1.5% gel electrophoresis. 16S rDNA amplicons were cleaned with AmpureXP beads (Agencourt, France) on a per-sample basis, then quantified using the Quant-iT Picogreen dsDNA Assay Kit (Invitrogen, Waltham, MA, USA). Amplicons were pooled evenly by DNA content and sequenced on an Illumina HiSeq (1 × 150 bp) at the Bauer Core Facility at Harvard University, generating 234,631 ± 110,737 (mean ± SD) sequences per sample passing filter (range: 75,898 to 391,101) (Table S1).

### 4.7. Microbiota Composition by 16S rRNA Gene Amplicon Analysis

Raw sequencing data were processed and analyzed using Quantitative Insights into Microbial Ecology 2 (QIIME2) [66]. Single-end sequences were first demultiplexed using the barcode sequences. The sequencing reads were then quality filtered, denoised, and merged using DADA2 [67] to generate the ASV feature table. For taxonomy classification, ASV feature sequences were aligned against the SILVA reference database [68]. Additional species level assignment to the NCBI RefSeq [69] 16S rRNA database supplemented by RDP [70] was accomplished using the *assignTaxonomy* and *addSpecies* functions of the DADA2 R package. 

### 4.8. Statistical Analysis

Microbial alpha and beta diversity measures were calculated at the ASV level using the vegan package in R. A principal coordinates analysis (PCoA) plot was generated with Bray–Curtis dissimilarity. Differences in microbiome compositions across different groups were tested by the permutational multivariate analysis of variance (PERMANOVA) using the “adonis” function in R’s vegan package. All PERMANOVA tests were performed with 9999 permutations based on Bray–Curtis dissimilarity. Differences between groups were analyzed using a Wilcoxon–Mann–Whitney test (unpaired) or Wilcoxon signed rank test (paired). The survival probability was computed by the Kaplan–Meier method.

MaAsLin2 [42] (multivariate association with linear model) was used for the adjustment of covariates when determining the significance of ASVs contributing to specific hematological variables and FI, while accounting for potentially confounding covariates. The linear mixed models included each mouse’s identifier as a random effect and other potential confounders as fixed effects. To be qualified for downstream analyses, an ASV feature needed to be detected in at least 10% of samples. The *p*-values were then adjusted using the Benjamini–Hochberg FDR method. The microbial features with corrected *q*-value < 0.2 were presented. For differential abundance analysis, we used ANCOM [49] (analysis of composition of microbiomes), with a Benjamini–Hochberg correction at 5% level of significance, and adjusted for cage, cohort, body mass, and diet. Only the ASVs that were presented in at least 10% of samples were included. To develop a model capable of predicting healthy aging, we implemented Elastic-net (ENET) using R’s caret package. A custom machine learning process was conducted using microbial features at 21 months of age to predict aging status at 30 months of age. We first trained our model with all microbial features. To further improve the biological plausibility, we then only included the microbial features significantly associated with FI. A total of 14 ASVs were selected based on the *q*-value (*q* < 0.2) from the MaAsLin2 model. Leave-one-out cross-validation (LOOCV) was applied with the *trainControl* function. To further validate our model, a null model was generated with randomly selected features (number of features = 14, times = 100). All statistical analyses were performed using R.

## Data Availability

Raw sequencing reads have been deposited in NCBI under accession number PRJNA739980.

## Supplementary Materials

The following are available online at https://www.mdpi.com/article/10.3390/nu13093290/s1, Figure S1: The effects of healthy aging on FI, body mass, and total bacterial load, Figure S2: The changes of body mass over time, Figure S3: The survival probability was computed by the Kaplan–Meier method, Figure S4: Impact of healthy aging on gut microbial communities, Figure S5: Relative abundance of aging-related microbial features in both normal and healthy aging mice, Figure S6: Relative abundance of aging-related microbial features, Figure S7: Relative abundance of healthy aging-related microbial features, Table S1: 16S rRNA gene sequencing metadata, Table S2: The effect of the aging process on blood cells in circulation, Table S3: The microbial features associated with blood markers identified by MaAsLin2, Table S4: The microbial features associated with the Frailty index identified by MaAsLin2, Table S5: Differentially abundant taxa between 21 and 30 months of age in healthy aging mice detected by ANCOM, adjusted for cage, cohort, and diet, Table S6: Differentially abundant taxa between 21 and 30 months of age in normal aging mice detected by ANCOM, adjusted for cage, cohort, and diet, Table S7: Differentially abundant taxa between 21 and 30 months of age detected by ANCOM, adjusted for cage, cohort, and diet, Table S8: Differentially abundant taxa between healthy and normal aging mice at 21 months of age detected by ANCOM, adjusted for cage, cohort, and diet, Table S9: Differentially abundant taxa between healthy and normal aging mice at 30 months of age detected by ANCOM, adjusted for cage, cohort, and diet.
