# Supplementary material for: Gut Microbiota Predicts Healthy Late-Life Aging in Male Mice
Source: Nutrients. 2021 Sep 21;13(9):3290. doi: 10.3390/nu13093290 (PMC8467910; doi:10.3390/nu13093290)
Supplement: Supplementary file 1 [file nutrients-13-03290-s001.zip › nutrients-1360718-supplementary.pdf]

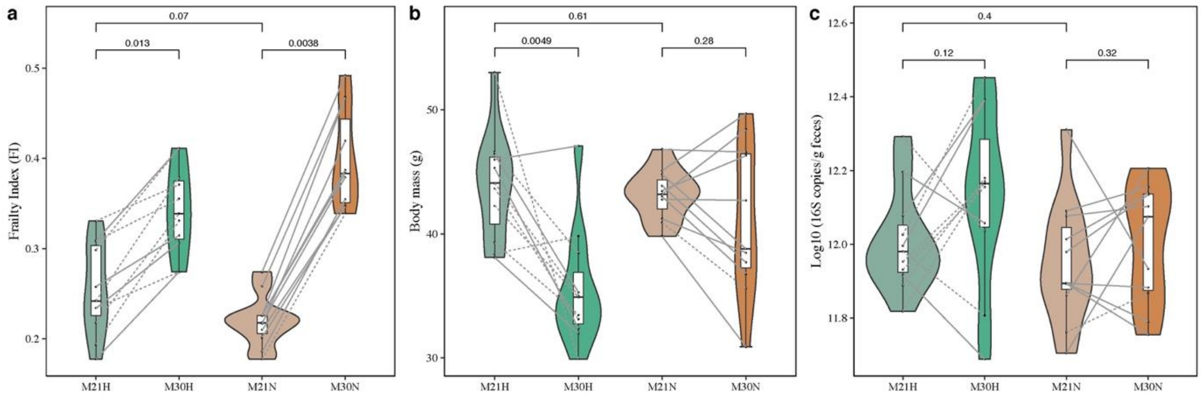

Figure S1. The effects of healthy aging on FI, body mass and total bacterial load. a, Frailty index changes with age. Mice were grouped into healthy and normal aging based on the median ΔFI at 30 months of age. **b**, Body mass changes with age. **c**, Total bacterial load changes with age. Points obtained for the same subject from 21 and 30 months of age are joined by solid (AL diet) and dotted (CR diet) lines. *P* value shown the results of Wilcoxon–Mann–Whitney test (unpaired) and Wilcoxon signed rank test (paired).

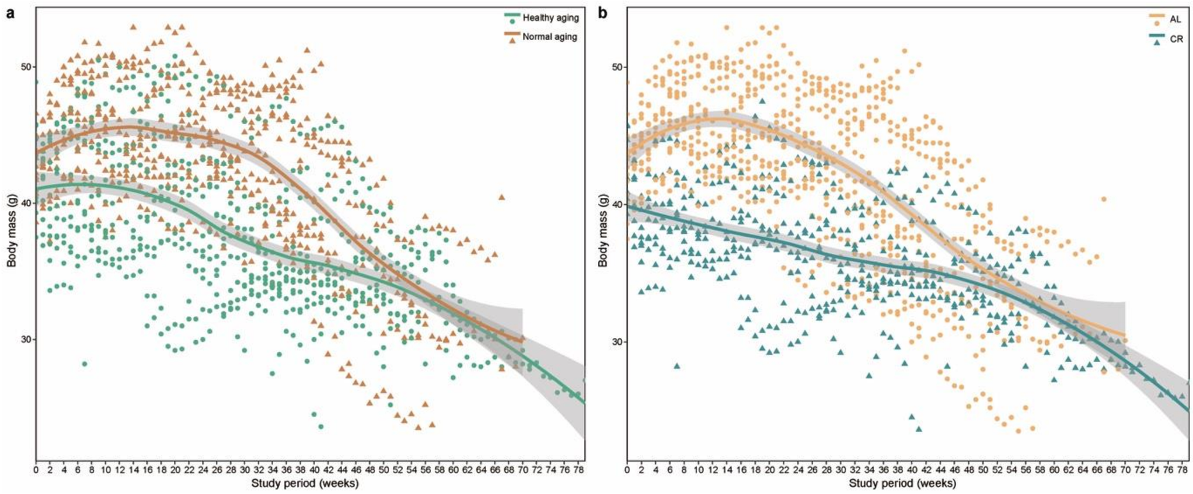

Figure S2. The changes of body mass over time. a, Healthy aging versus Normal aging mice. **b**, AL diet versus CR diet. Curves show LOESS fit for the data per category, and shaded areas show 95% confidence intervals for the fit.

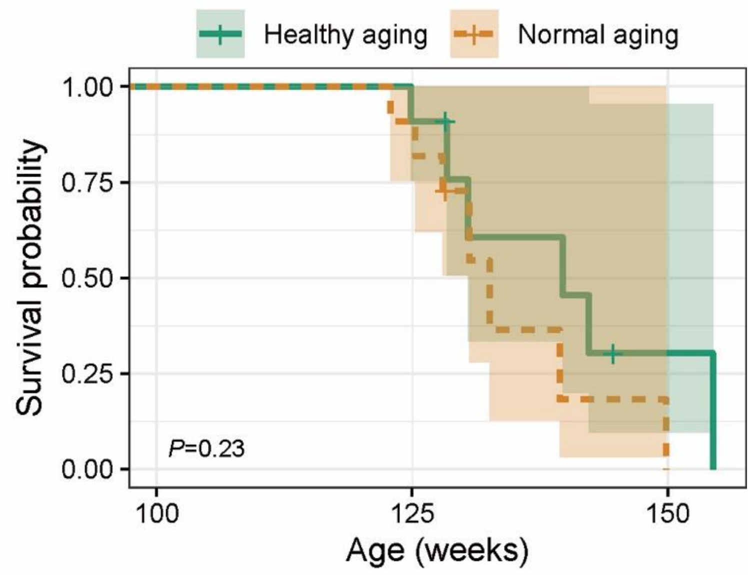

Figure S3. The survival probability was computed by the Kaplan-Meier method. *P* value is the result of log-rank test.

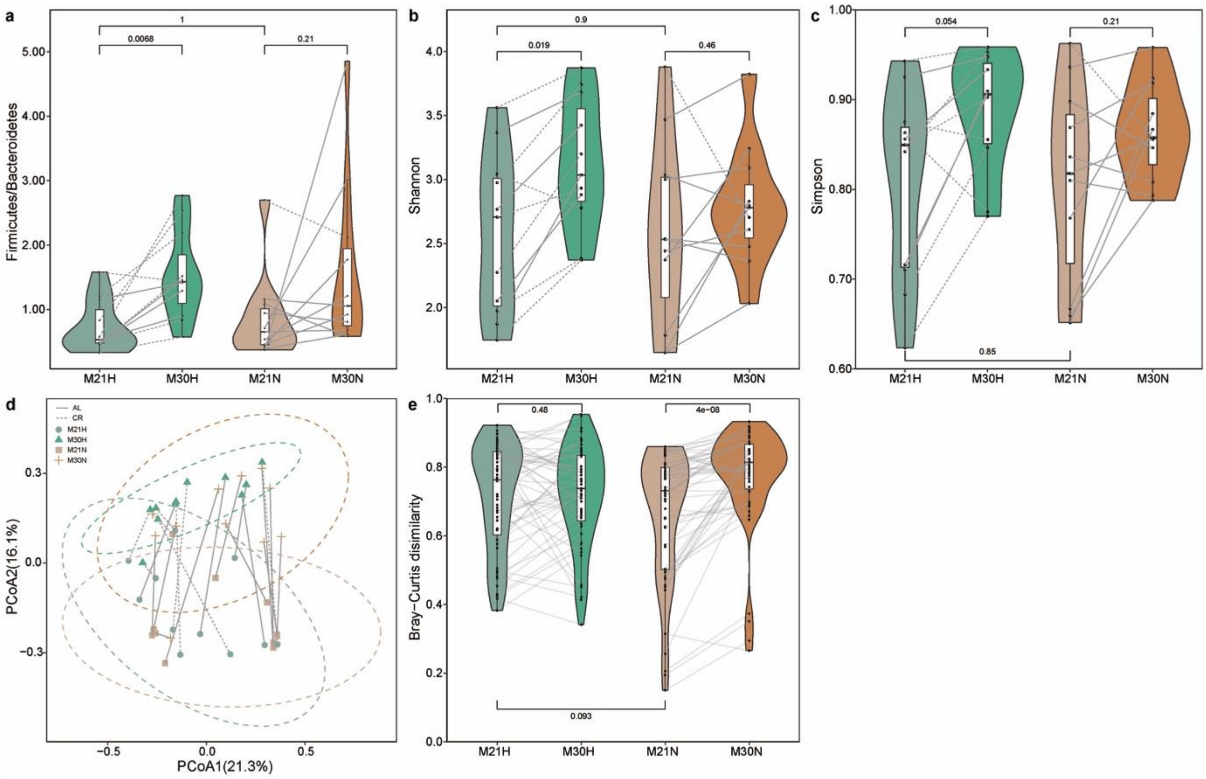

Figure S4. Impact of healthy aging on gut microbial communities. a, The ratio of Firmicutes to Bacteroidetes. Alpha diversity using Shannon (**b**) and Simpson (**c**) index. **d**, Beta diversity using Principal Coordinate Analysis (PCoA) of Bray–Curtis dissimilarity. The dotted ellipse borders with color represent the 95% confidence interval. **e**, Boxplot of gut microbiome Bray–Curtis dissimilarity between subjects within each group. Mice were grouped into healthy and normal aging based on the median ΔFI at 30 months of age. Points obtained for the same subject from 21 and 30 months of age in **a-d** are joined by solid (AL

diet) and dotted (CR diet) lines. Points obtained for the same subject pairs from 21 and 30 months of age in **e** are joined by solid line. *P* value shown are the result of Wilcoxon–Mann–Whitney test (unpaired) and Wilcoxon signed rank test (paired).

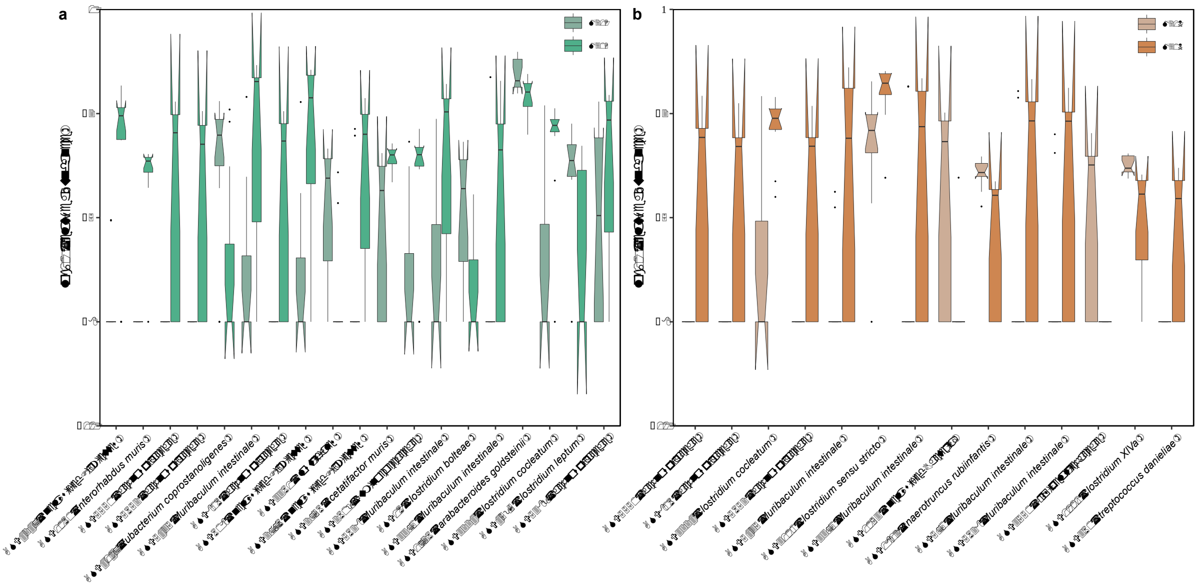

Figure S5. Relative abundance of aging related microbial features in both normal and healthy aging mice. The differential abundant ASVs that differed significantly between 21 and 30months of age for healthy (**a**) and normal (**b**) aging mice identified by analysis of composition of microbiomes (ANCOM). The model was simultaneously adjusted for potential confounders including cage, cohort, diet, and body mass. Mice were grouped into healthy and normal aging based on the median ΔFI at 30 months of age. The top differentially abundant taxa were ranked based on their W statistics (a high "w score" generated by this test indicates the greater likelihood that the null hypothesis can be rejected, indicating the number of times a parameter is significantly different between groups) (from left to right). The relative abundance (%) are plotted on log10 scale. The notches in the boxplots show the 95% confidence interval around the median.

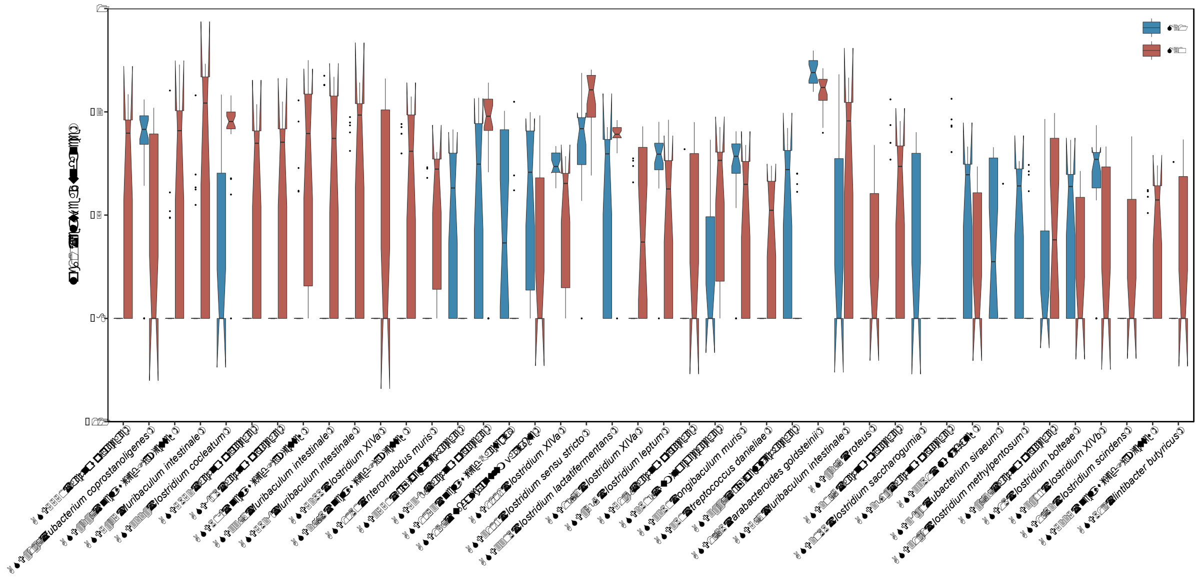

Figure S6. Relative abundance of aging related microbial features. The differential abundant ASVs that differed significantly between 21 and 30months of age identified by ANCOM. The model was simultaneously adjusted for potential confounders including cage, cohort, diet, and body mass. The top differentially abundant taxa were ranked based on their W statistics (a high “w score” generated by this test indicates the greater likelihood that the null hypothesis can be rejected, indicating the number of times a parameter is significantly different between groups) (from left to right). The relative abundance (%) are plotted on log10 scale. The notches in the boxplots show the 95% confidence interval around the median.

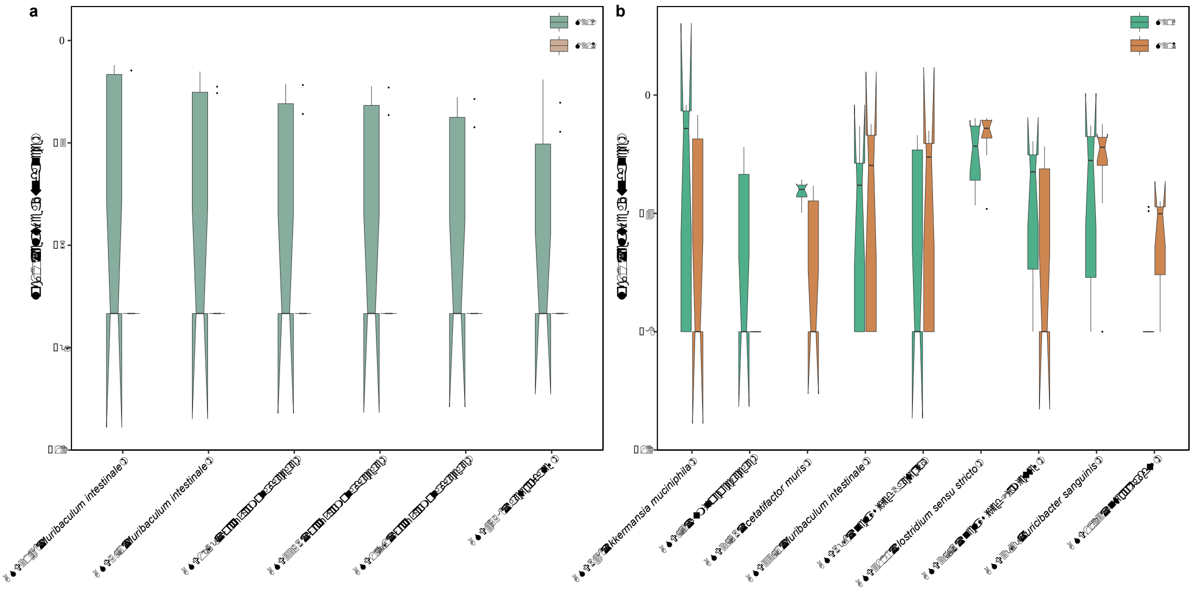

Figure S7 Relative abundance of healthy aging related microbial features. The differential abundant ASVs that differed significantly between healthy and normal aging mice at 21 (**a**) and 30 (**b**) months of ages identified by ANCOM. The model was simultaneously adjusted for potential confounders including cage, cohort, diet, and body

mass. Mice were grouped into healthy and normal aging based on the median ΔFI at 30 months of age. The top differentially abundant taxa were ranked based on their W statistics (a high "w score" generated by this test indicates the greater likelihood that the null hypothesis can be rejected, indicating the number of times a parameter is significantly different between groups) (from left to right). The relative abundance (%) are plotted on log10 scale. The notches in the boxplots show the 95% confidence interval around the median.

Table S1. 16S rRNA gene sequencing metadata.

Sample ID	Mouse ID	Time point	Diet	Barcode-sequence	Raw sequence count	Final sequence count	Number of ASVs	Aging Status
YY001	A-1	M21	AL	GGATACTCGCAT	154570	135037	106	Normal aging
YY004	A-1	M30	AL	ACTAGACGACTA	415374	352401	157	Normal aging
YY010	A-21	M21	AL	CTTGTGCGACAA	439383	373296	216	Normal aging
YY012	A-21	M30	AL	AGGTTAAGTGCT	422939	338440	250	Normal aging
YY013	A-23	M21	AL	ATGCTCTAGAGA	390427	324117	239	Healthy aging
YY015	A-23	M30	AL	CGATTTAGGCCA	299124	248027	240	Healthy aging
YY016	A-24	M21	AL	GGTACAATGATC	449154	380134	288	Normal aging
YY018	A-24	M30	AL	AGAGTAAGCCGG	382718	313578	229	Normal aging
YY019	A-26	M21	CR	GACACTCACCGT	371088	310319	300	Healthy aging
YY021	A-26	M30	CR	AGCTAGCGTTCA	353458	290435	292	Healthy aging
YY022	A-27	M21	CR	TCTTCTGCCCTA	371714	306639	293	Normal aging
YY024	A-27	M30	CR	ACTGTCGCAGTA	465584	388876	214	Normal aging
YY025	A-28	M21	CR	CTGATGTACACG	424321	351496	311	Healthy aging
YY027	A-28	M30	CR	TCAGAGTAGACT	393168	311260	305	Healthy aging
YY045	A-81	M21	AL	GTTCAGACTAGC	338153	269337	176	Normal aging
YY053	A-101	M21	AL	ACTAATACGCGA	435557	391101	160	Healthy aging
YY063	A-281	M21	AL	ACGAGGAGTCGA	415094	335968	226	Normal aging
YY066	A-284	M21	AL	ACATCCCTACTT	394919	322213	235	Healthy aging
YY067	A-289	M21	CR	CCTTAAGGGCAT	438364	364232	177	Healthy aging
YY068	A-290	M21	CR	TTCGTGAGGATA	418761	348879	156	Healthy aging
YY071	A-297	M21	AL	GCGGTACTACTA	376527	320687	208	Normal aging
YY072	A-298	M21	AL	TCGTTCAGGACC	441041	365541	195	Normal aging
YY073	A-300	M21	AL	CTTCTTCGCCCT	419897	352836	219	Normal aging
YY079	A-306	M21	CR	TCAGCTGACTAG	414372	337781	229	Healthy aging
YY097	A-81	M30	AL	AGTCGAACGAGG	125864	113019	149	Normal aging

YY099	A-101	M30	AL	TGCAGTCCTCGA	143687	128409	157	Healthy aging
YY101	A-161	M21	AL	GTGGAGTCTCAT	164126	160052	150	Normal aging
YY103	A-161	M30	AL	GCGTTCTAGCTG	152005	126296	148	Normal aging
YY104	A-164	M21	AL	GCTGTACGGATT	113804	110673	128	Healthy aging
YY106	A-164	M30	AL	AGTCGTGCACAT	123834	110799	116	Healthy aging
YY107	A-165	M21	CR	ACCATAGCTCCG	132298	124562	152	Healthy aging
YY109	A-165	M30	CR	GCTCGAAGATTC	156099	141296	131	Healthy aging
YY110	A-166	M21	CR	TAGGCATGCTTG	179628	174312	153	Healthy aging
YY112	A-166	M30	CR	ATCACCAGGTGT	109369	101016	118	Healthy aging
YY118	A-184	M21	AL	GAGATCGCCTAT	147393	142804	137	Normal aging
YY120	A-184	M30	AL	TGGTCAACGATA	91525	83645	95	Normal aging
YY126	A-281	M30	AL	ATTCTGCCGAAG	126405	98450	201	Normal aging
YY130	A-284	M30	AL	CAAATTCGGGAT	83711	75898	202	Healthy aging
YY133	A-289	M30	CR	ACTTCCAACTTC	111893	96359	134	Healthy aging
YY136	A-290	M30	CR	GTCGTGTAGCCT	117763	114010	176	Healthy aging
YY142	A-297	M30	AL	GTACGATATGAC	199365	179853	161	Normal aging
YY145	A-298	M30	AL	CCAATACGCCTG	122230	113580	146	Normal aging
YY148	A-300	M30	AL	TGTCGCAAATAG	151897	136678	141	Normal aging
YY151	A-306	M30	CR	TGTAACGCCGAT	178578	159425	211	Healthy aging

Table S2. The effect of aging process on blood cells in circulation. The data was shown as mean ± standard deviation. *P* value shown the results of Wilcoxon–Mann–Whitney test (unpaired) and Wilcoxon signed rank test (paired) adjusted using the Benjamini–Hochberg FDR method. WBC: White blood cell, NE: Neutrophils count, LY: Lymphocytes count, MO: Monocytes count, EO: Eosinophils count, BA: Basophils count, NEp: Neutrophils percentage, LYp: Lymphocytes percentage, MOp: Monocytes percentage, EOp: Eosinophils percentage, BA: Basophils percentage, RBC: Red blood cell count, Hb: Hemoglobin, HCT: Hematocrit, MCV: Mean corpuscular volume, MCH: Mean corpuscular hemoglobin, MCHC: Mean corpuscular hemoglobin concentration, RDW: Red cell distribution width, PLT: Platelet count, MPV: Mean platelet volume, NLR: Neutrophils to Lymphocytes ratio.

	**M21**			**M30**			**Wilcoxon test**	**Wilcoxon–Mann–Whitney test**					
Blood markers	M21 (n=22)	M21_H (n=11)	M21_N (n=11)	M30 (n=19)	M30_H (n=10)	M30_N (n=9)	M21_H vs M21_N	M21 vs M30	M21_H vs M30_H	M21_N vs M30_N	M30_H vs M30_N	M21 vs M30_H	M21 vs M30_N

WBC	9.830 ± 2.413	10.273 ± 2.034	9.387 ± 2.768	6.441 ± 4.273	6.814 ± 4.291	6.027 ± 4.472	0.966	0.022	0.177	0.157	0.746	0.123	0.046
NE	1.831 ± 0.63	2.021 ± 0.645	1.641 ± 0.581	2.183 ± 1.936	2.076 ± 1.438	2.301 ± 2.465	0.839	0.699	0.578	0.97	0.941	0.871	0.792
LY	7.756 ± 1.953	7.993 ± 1.565	7.52 ± 2.332	4.328 ± 2.691	5.135 ± 2.518	3.432 ± 2.729	0.943	0.001	0.059	0.017	0.469	0.033	0.004
MO	0.234 ± 0.082	0.25 ± 0.099	0.218 ± 0.061	0.271 ± 0.199	0.261 ± 0.162	0.282 ± 0.243	0.839	0.917	0.874	0.873	0.967	0.871	1
EO	0.008 ± 0.014	0.005 ± 0.009	0.011 ± 0.018	0.026 ± 0.071	0.041 ± 0.096	0.01 ± 0.019	0.839	0.443	0.42	0.97	0.709	0.348	0.866
BA	0.001 ± 0.003	0 ± 0	0.002 ± 0.004	0.001 ± 0.002	0 ± 0	0.001 ± 0.003	0.839	0.699	NA	0.873	0.6	0.441	0.944
NEp	18.582 ± 3.969	19.552 ± 4.005	17.613 ± 3.87	33.034 ± 15.552	27.383 ± 8.807	39.312 ± 19.305	0.839	0.001	0.059	0.016	0.51	0.009	0.005
LYp	78.881 ± 4.36	77.948 ± 4.609	79.814 ± 4.093	61.905 ± 17.272	68.685 ± 9.708	54.372 ± 21.069	0.839	0.001	0.059	0.016	0.51	0.009	0.005
MOp	2.439 ± 0.804	2.422 ± 0.775	2.456 ± 0.87	4.69 ± 3.031	3.467 ± 1.208	6.049 ± 3.884	0.843	0.002	0.12	0.016	0.414	0.07	0.004
EOp	0.081 ± 0.113	0.063 ± 0.08	0.099 ± 0.14	0.312 ± 0.593	0.432 ± 0.784	0.179 ± 0.247	0.839	0.339	0.474	0.777	0.967	0.378	0.56
BAp	0.015 ± 0.019	0.012 ± 0.011	0.018 ± 0.024	0.046 ± 0.062	0.032 ± 0.045	0.061 ± 0.077	0.843	0.502	0.874	0.542	0.709	0.871	0.391
RBC	8.808 ± 1.217	9.08 ± 0.438	8.535 ± 1.66	7.608 ± 1.697	7.935 ± 0.742	7.246 ± 2.361	0.839	0.002	0.012	0.196	0.6	0.009	0.053
Hb	10.855 ± 1.698	11.273 ± 1.07	10.436 ± 2.128	9.837 ± 1.649	10.29 ± 0.61	9.333 ± 2.27	0.843	0.023	0.059	0.18	0.429	0.123	0.053
HCT	37.518 ± 5.18	38.882 ± 1.982	36.155 ± 6.952	35.304 ± 6.621	35.647 ± 3.047	34.922 ± 9.374	0.839	0.039	0.059	0.542	0.709	0.07	0.236
MCV	59.432 ± 79.046	76.473 ± 111.721	42.391 ± 0.89	47.111 ± 5.321	45.17 ± 4.29	49.267 ± 5.75	0.839	0.005	0.316	0.016	0.467	0.084	0.009
MCH	12.332 ± 0.978	12.409 ± 0.836	12.255 ± 1.139	13.126 ± 1.163	13.04 ± 0.9	13.222 ± 1.453	0.881	0.064	0.263	0.24	0.967	0.123	0.236
MCHC	28.932 ± 2.096	28.973 ± 1.826	28.891 ± 2.427	28.053 ± 2.756	29.01 ± 2.87	26.989 ± 2.323	0.919	0.396	0.874	0.18	0.383	0.871	0.083
RDW	17.532 ± 0.764	17.655 ± 0.636	17.409 ± 0.888	19.621 ± 2.842	18.93 ± 2.155	20.389 ± 3.42	0.839	0.037	0.459	0.065	0.668	0.189	0.053

PLT	1390.455 ± 254.416	1470.727 ± 253.589	1310.182 ± 239.676	1561.263 ± 418.144	1777.1 ± 314.232	1321.444 ± 399.227	0.839	0.234	0.063	0.873	0.226	0.009	0.56
MPV	5.182 ± 0.168	5.2 ± 0.126	5.164 ± 0.206	5.537 ± 0.527	5.26 ± 0.19	5.844 ± 0.619	0.843	0.02	0.578	0.017	0.226	0.348	0.005
NLR	0.239 ± 0.065	0.255 ± 0.068	0.224 ± 0.061	0.693 ± 0.646	0.425 ± 0.205	0.991 ± 0.837	0.839	0.001	0.059	0.016	0.51	0.009	0.005

Table S3. The microbial features associated with blood markers identified by MaAsLin2. The relative abundance (%) was shown as mean ± standard deviation.

**ASVs**	**Taxonomy**	**Relative abundance (%)**
ASV890	Ruminococcaceae	0.004 ± 0.011
ASV806	Lachnospiraceae	0.166 ± 0.394
ASV5690	*Flavonifractor plautii*	0.242 ± 0.397
ASV5652	Lachnospiraceae	0.153 ± 0.355
ASV5625	Unclassified Firmicutes	0.054 ± 0.234
ASV5550	Lachnospiraceae	0.292 ± 0.671
ASV555	*Acetatifactor muris*	0.002 ± 0.005
ASV5396	*Acetatifactor muris*	0.025 ± 0.079
ASV5138	Unclassified Proteobacteria	0.108 ± 0.321
ASV4558	Bacteroidales	0.355 ± 1.284
ASV3949	*Anaerotruncus*	0.010 ± 0.018
ASV3897	Unclassified Bacteria	0.019 ± 0.056
ASV3729	*Clostridium aldenense*	0.003 ± 0.010
ASV3535	*Muribaculum intestinale*	0.084 ± 0.226
ASV2973	*Intestinimonas butyriciproducens*	0.006 ± 0.016
ASV2878	Lachnospiraceae	0.020 ± 0.064
ASV2868	*Oscillibacter*	0.007 ± 0.024
ASV2733	*Clostridium XlVa*	0.048 ± 0.154
ASV2710	Unclassified Firmicutes	0.001 ± 0.002
ASV2261	Ruminococcaceae	0.001 ± 0.003
ASV1983	Ruminococcaceae	0.043 ± 0.085
ASV1970	*Clostridium XlVa*	0.036 ± 0.077
ASV1513	Lachnospiraceae	0.002 ± 0.002
ASV1466	Unclassified Firmicutes	0.005 ± 0.018

Table S4. The microbial features associated with Frailty index identified by MaAsLin2. The relative abundance (%) was shown as mean ± standard deviation.

ASVs	Taxonomy	Relative abundance (%)
ASV3100	*Clostridium sensu stricto*	4.148 ± 5.608
ASV2882	*Clostridium XlVa*	0.108 ± 0.133
ASV847	*Phocea massiliensis*	0.004 ± 0.008
ASV338	Lachnospiraceae	0.011 ± 0.039
ASV1726	*Parabacteroides goldsteinii*	13.017 ± 13.852
ASV5389	Lachnospiraceae	1.061 ± 1.702
ASV1123	*Enterorhabdus*	0.018 ± 0.025
ASV1101	*Clostridium XlVa*	0.025 ± 0.025
ASV807	Bacteria	0.001 ± 0.003
ASV742	Lachnospiraceae	0.010 ± 0.033
ASV157	*Subdoligranulum variabile*	0.131 ± 0.243
ASV232	Ruminococcaceae	0.009 ± 0.014
ASV2980	Lachnospiraceae	0.101 ± 0.220
ASV466	Lachnospiraceae	0.028 ± 0.080

Table S5. Differentially abundant taxa between 21 and 30 months of age in healthy aging mice detected by ANCOM, adjusted for cage, cohort and diet. For each ASV, the first column represents its taxonomy information, the second column represents its W score and subsequent four columns represent logical indicators of whether it is differentially abundant under a series of cutoffs (0.9, 0.8, 0.7, and 0.6, a prevalence cutoff on the entire set of ASVs). The last two columns denote its relative abundance (%) in each group shown as mean ± standard deviation.

**ASVs**	**Taxonomy**	**W_score**	**detected_0.9**	**detected_0.8**	**detected_0.7**	**detected_0.6**	**M21H**	**M30H**
ASV4247	Unclassified Firmicutes	368	TRUE	TRUE	TRUE	TRUE	0 ± 0	1.357 ± 1.883
ASV1060	*Enterorhabdus muris*	352	TRUE	TRUE	TRUE	TRUE	0 ± 0	0.039 ± 0.023
ASV5550	Lachnospiraceae	350	TRUE	TRUE	TRUE	TRUE	0 ± 0	0.631 ± 0.797
ASV5652	Lachnospiraceae	345	TRUE	TRUE	TRUE	TRUE	0 ± 0	0.331 ± 0.44
ASV4147	*Eubacterium coprostanoligenes*	331	FALSE	TRUE	TRUE	TRUE	0.622 ± 0.836	0.174 ± 0.414
ASV5435	*Muribaculum intestinale*	330	FALSE	TRUE	TRUE	TRUE	0.275 ± 0.906	7.51 ± 8.01
ASV806	Lachnospiraceae	326	FALSE	TRUE	TRUE	TRUE	0 ± 0	0.345 ± 0.446
ASV608	Unclassified Firmicutes	324	FALSE	TRUE	TRUE	TRUE	0.196 ± 0.648	5.741 ± 7.033
ASV3361	Clostridiales	277	FALSE	FALSE	TRUE	TRUE	0.027 ± 0.032	0.002 ± 0.006
ASV2776	Unclassified Firmicutes	275	FALSE	FALSE	TRUE	TRUE	0.054 ± 0.123	0.74 ± 1.023
ASV2756	*Acetatifactor muris*	261	FALSE	FALSE	FALSE	TRUE	0.017 ± 0.024	0.069 ± 0.042

ASV2609	Ruminococcaceae	256	FALSE	FALSE	FALSE	TRUE	0.018 ±0.046	0.085 ±0.1
ASV5628	*Muribaculum intestinale*	254	FALSE	FALSE	FALSE	TRUE	0.145 ±0.258	1.845 ±2.288
ASV16	*Clostridium bolteae*	253	FALSE	FALSE	FALSE	TRUE	0.034 ±0.049	0.001 ±0.002
ASV3370	*Muribaculum intestinale*	250	FALSE	FALSE	FALSE	TRUE	1.013 ±3.359	1.042 ±2.593
ASV1726	*Parabacteroides goldsteinii*	248	FALSE	FALSE	FALSE	TRUE	20.989 ±20.721	5.247 ±4.615
ASV3224	*Clostridium cocleatum*	246	FALSE	FALSE	FALSE	TRUE	0.275 ±0.555	0.558 ±0.461
ASV4595	*Clostridium leptum*	234	FALSE	FALSE	FALSE	TRUE	0.121 ±0.169	0.024 ±0.04
ASV5389	Lachnospiraceae	229	FALSE	FALSE	FALSE	TRUE	0.369 ±0.731	1.192 ±1.241

Table S6. Differentially abundant taxa between 21 and 30 months of age in normal aging mice detected by ANCOM, adjusted for cage, cohort and diet. For each ASV, the first column represents its taxonomy information, the second column represents its W score and subsequent four columns represent logical indicators of whether it is differentially abundant under a series of cutoffs (0.9, 0.8, 0.7, and 0.6, a prevalence cutoff on the entire set of ASVs). The last two columns denote its relative abundance (%) in each group shown as mean ±standard deviation.

**ASVs**	**Taxonomy**	**W_score**	**detected_0.9**	**detected_0.8**	**detected_0.7**	**detected_0.6**	**M21N**	**M30N**
ASV5550	Lachnospiraceae	334	TRUE	TRUE	TRUE	TRUE	0 ±0	0.477 ±0.938
ASV806	Lachnospiraceae	327	TRUE	TRUE	TRUE	TRUE	0 ±0	0.283 ±0.576
ASV3224	*Clostridium cocleatum*	327	TRUE	TRUE	TRUE	TRUE	0.48 ±1.005	0.955 ±0.931
ASV5652	Lachnospiraceae	326	TRUE	TRUE	TRUE	TRUE	0 ±0	0.248 ±0.481
ASV5435	*Muribaculum intestinale*	316	FALSE	TRUE	TRUE	TRUE	0.001 ±0.002	4.381 ±7.109
ASV3100	*Clostridium sensu stricto*	296	FALSE	TRUE	TRUE	TRUE	1.222 ±2.458	8.285 ±6.248
ASV3370	*Muribaculum intestinale*	285	FALSE	FALSE	TRUE	TRUE	1.091 ±2.427	2.785 ±3.561
ASV1053	Unclassified Bacteria	278	FALSE	FALSE	TRUE	TRUE	0.331 ±0.39	0.001 ±0.004
ASV1812	*Anaerotruncus rubiinfantis*	263	FALSE	FALSE	TRUE	TRUE	0.025 ±0.017	0.004 ±0.004
ASV570	*Muribaculum intestinale*	258	FALSE	FALSE	TRUE	TRUE	0.665 ±1.52	1.999 ±3.191
ASV5628	*Muribaculum intestinale*	254	FALSE	FALSE	TRUE	TRUE	0.03 ±0.077	1 ±1.592
ASV3550	Erysipelotrichaceae	250	FALSE	FALSE	FALSE	TRUE	0.052 ±0.079	0 ±0
ASV1101	*Clostridium XlVa*	238	FALSE	FALSE	FALSE	TRUE	0.038 ±0.023	0.007 ±0.006
ASV360	*Streptococcus danieliae*	220	FALSE	FALSE	FALSE	TRUE	0 ±0	0.008 ±0.01

Table S7. Differentially abundant taxa between 21 and 30 months of age detected by ANCOM, adjusted for cage, cohort and diet. For each ASV, the first column represents its taxonomy information, the second column represents its W score and subsequent four columns represent logical indicators of whether it is differentially abundant under a series of cutoffs (0.9, 0.8, 0.7, and 0.6, a prevalence cutoff on the entire set of ASVs). The last two

columns denote its relative abundance (%) in each group shown as mean ±standard deviation.

**ASVs**	**Taxonomy**	**W_score**	**detected_0.9**	**detected_0.8**	**detected_0.7**	**detected_0.6**	**M21**	**M30**
ASV5550	Lachnospiraceae	375	TRUE	TRUE	TRUE	TRUE	0 ±0	0.554 ±0.853
ASV4147	*Eubacterium coprostanoligenes*	372	TRUE	TRUE	TRUE	TRUE	0.55 ±0.661	0.178 ±0.332
ASV4247	Unclassified Firmicutes	369	TRUE	TRUE	TRUE	TRUE	0.189 ±0.886	1.879 ±5.061
ASV5435	*Muribaculum intestinale*	369	TRUE	TRUE	TRUE	TRUE	0.138 ±0.641	5.946 ±7.562
ASV3224	*Clostridium cocleatum*	366	TRUE	TRUE	TRUE	TRUE	0.377 ±0.798	0.756 ±0.745
ASV5652	Lachnospiraceae	366	TRUE	TRUE	TRUE	TRUE	0 ±0	0.29 ±0.452
ASV806	Lachnospiraceae	365	TRUE	TRUE	TRUE	TRUE	0 ±0	0.314 ±0.504
ASV608	Unclassified Firmicutes	360	TRUE	TRUE	TRUE	TRUE	0.123 ±0.466	4.633 ±8.181
ASV3370	*Muribaculum intestinale*	357	TRUE	TRUE	TRUE	TRUE	1.052 ±2.86	1.914 ±3.168
ASV5628	*Muribaculum intestinale*	354	TRUE	TRUE	TRUE	TRUE	0.087 ±0.195	1.422 ±1.971
ASV5266	*Clostridium XlVa*	342	FALSE	TRUE	TRUE	TRUE	0 ±0	1.084 ±2.245
ASV2776	Unclassified Firmicutes	341	FALSE	TRUE	TRUE	TRUE	0.05 ±0.125	0.534 ±0.842
ASV1060	*Enterorhabdus muris*	340	FALSE	TRUE	TRUE	TRUE	0.003 ±0.007	0.025 ±0.023
ASV3550	Erysipelotrichaceae	332	FALSE	TRUE	TRUE	TRUE	0.056 ±0.093	0 ±0
ASV5389	Lachnospiraceae	327	FALSE	TRUE	TRUE	TRUE	0.432 ±0.738	1.69 ±2.134
ASV1053	Unclassified Bacteria	324	FALSE	TRUE	TRUE	TRUE	0.224 ±0.348	0.09 ±0.417
ASV157	*Subdoligranulum variabile*	318	FALSE	TRUE	TRUE	TRUE	0.196 ±0.284	0.066 ±0.176
ASV1101	*Clostridium XlVa*	318	FALSE	TRUE	TRUE	TRUE	0.039 ±0.027	0.012 ±0.013
ASV3100	*Clostridium sensu stricto*	318	FALSE	TRUE	TRUE	TRUE	1.776 ±3.757	6.518 ±6.203
ASV3306	*Clostridium lactatifermentans*	316	FALSE	TRUE	TRUE	TRUE	0.098 ±0.112	0.261 ±0.154
ASV1970	*Clostridium XlVa*	312	FALSE	TRUE	TRUE	TRUE	0.005 ±0.013	0.071 ±0.103
ASV4595	*Clostridium leptum*	310	FALSE	TRUE	TRUE	TRUE	0.102 ±0.126	0.055 ±0.129
ASV5149	Lachnospiraceae	310	FALSE	TRUE	TRUE	TRUE	0.004 ±0.017	0.046 ±0.109
ASV2609	Ruminococcaceae	308	FALSE	TRUE	TRUE	TRUE	0.02 ±0.043	0.058 ±0.078
ASV3260	*Longibaculum muris*	308	FALSE	TRUE	TRUE	TRUE	0.068 ±0.07	0.025 ±0.033
ASV360	*Streptococcus danieliae*	305	FALSE	FALSE	TRUE	TRUE	0 ±0	0.006 ±0.009
ASV3447	Erysipelotrichaceae	300	FALSE	FALSE	TRUE	TRUE	0.058 ±0.096	0.002 ±0.005
ASV1726	*Parabacteroides goldsteinii*	299	FALSE	FALSE	TRUE	TRUE	19.963 ± 16.342	6.07 ±4.97
ASV570	*Muribaculum intestinale*	292	FALSE	FALSE	TRUE	TRUE	1.52 ±3.134	1.592 ±2.524
ASV4275	*Proteus*	284	FALSE	FALSE	TRUE	TRUE	0 ±0	0.01 ±0.025
ASV2075	Lachnospiraceae	283	FALSE	FALSE	TRUE	TRUE	0.131 ±0.492	0.097 ±0.154

ASV2066	*Clostridium saccharogumia*	280	FALSE	FALSE	TRUE	TRUE	0.05 ±0.084	0 ±0
ASV2904	Lachnospiraceae	278	FALSE	FALSE	TRUE	TRUE	0 ±0	0.174 ±0.52
ASV3361	Clostridiales	274	FALSE	FALSE	TRUE	TRUE	0.021 ±0.025	0.004 ±0.007
ASV3840	*Eubacterium siraeum*	272	FALSE	FALSE	TRUE	TRUE	0.023 ±0.033	0 ±0.002
ASV3141	*Clostridium methylpentosum*	270	FALSE	FALSE	TRUE	TRUE	0.012 ±0.013	0.003 ±0.008
ASV4737	Lachnospiraceae	267	FALSE	FALSE	FALSE	TRUE	0.059 ±0.173	0.159 ±0.275
ASV16	*Clostridium bolteae*	253	FALSE	FALSE	FALSE	TRUE	0.02 ±0.037	0.002 ±0.005
ASV3400	*Clostridium XlVb*	253	FALSE	FALSE	FALSE	TRUE	0.068 ±0.098	0.017 ±0.026
ASV1762	*Clostridium scindens*	249	FALSE	FALSE	FALSE	TRUE	0 ±0	0.022 ±0.047
ASV5225	Unclassified Firmicutes	244	FALSE	FALSE	FALSE	TRUE	0.001 ±0.002	0.006 ±0.007
ASV613	*Flintibacter butyricus*	232	FALSE	FALSE	FALSE	TRUE	0.002 ±0.007	0.015 ±0.036

Table S8. Differentially abundant taxa between healthy and normal aging mice at 21 months of age detected by ANCOM, adjusted for cage, cohort and diet. For each ASV, the first column represents its taxonomy information, the second column represents its W score and subsequent four columns represent logical indicators of whether it is differentially abundant under a series of cutoffs (0.9, 0.8, 0.7, and 0.6, a prevalence cutoff on the entire set of ASVs). The last two columns denote its relative abundance (%) in each group shown as mean ±standard deviation.

ASVs	Taxonomy	W_score	detected_ 0.9	detected_ 0.8	detected_ 0.7	detected_ 0.6	Healthy aging (M21H)	Normal aging (M21N)
ASV2048	*Muribaculum intestinale*	366	TRUE	TRUE	TRUE	TRUE	5.129 ±7.579	1.2 ±3.98
ASV570	*Muribaculum intestinale*	289	FALSE	FALSE	TRUE	TRUE	2.375 ±4.087	0.665 ±1.52
ASV1959	Porphyromonadaceae	268	FALSE	FALSE	TRUE	TRUE	0.982 ±1.702	0.513 ±1.483
ASV3256	Porphyromonadaceae	260	FALSE	FALSE	FALSE	TRUE	0.841 ±1.441	0.44 ±1.258
ASV1791	Porphyromonadaceae	238	FALSE	FALSE	FALSE	TRUE	0.395 ±0.688	0.202 ±0.58
ASV4558	Bacteroidales	232	FALSE	FALSE	FALSE	TRUE	1.063 ±2.401	0.156 ±0.452

Table S9. Differentially abundant taxa between healthy and normal aging mice at 30 months of age detected by ANCOM, adjusted for cage, cohort and diet. For each ASV, the first column represents its taxonomy information, the second column represents its W score and subsequent four columns represent logical indicators of whether it is differentially abundant under a series of cutoffs (0.9, 0.8, 0.7, and 0.6, a prevalence cutoff on the entire set of ASVs). The last two columns denote its relative abundance (%) in each group shown as mean ±standard deviation.

ASVs	Taxonomy	W_score	detected_0.9	detected_0.8	detected_0.7	detected_0.6	Healthy aging (M30H)	Normal aging (M30N)
ASV648	*Akkermansia muciniphila*	323	TRUE	TRUE	TRUE	TRUE	15.487 ±18.623	3.812 ±6.979
ASV73	Ruminococcaceae	300	FALSE	TRUE	TRUE	TRUE	0.298 ±0.566	0 ±0
ASV2756	*Acetatifactor muris*	270	FALSE	FALSE	TRUE	TRUE	0.069 ±0.042	0.02 ±0.033
ASV3370	*Muribaculum intestinale*	258	FALSE	FALSE	TRUE	TRUE	1.042 ±2.593	2.785 ±3.561
ASV698	Unclassified Bacteria	253	FALSE	FALSE	TRUE	TRUE	0.935 ±1.527	1.547 ±2.031
ASV3100	Clostridium sensu stricto	248	FALSE	FALSE	TRUE	TRUE	4.75 ±5.907	8.285 ±6.248
ASV2776	Unclassified Firmicutes	228	FALSE	FALSE	FALSE	TRUE	0.74 ±1.023	0.329 ±0.591
ASV3939	*Turicibacter sanguinis*	218	FALSE	FALSE	FALSE	TRUE	2.442 ±3.116	2.59 ±3.045
ASV1123	*Enterorhabdus*	216	FALSE	FALSE	FALSE	TRUE	0.003 ±0.006	0.011 ±0.009
